# Unresectable Undifferentiated Embryonal Sarcoma of the Liver in an Adult Male Treated with Chemotherapy and Orthotopic Liver Transplantation

**DOI:** 10.7759/cureus.1759

**Published:** 2017-10-08

**Authors:** Zarak H Khan, Kamran Ilyas, Hamza H Khan, Haider Ghazanfar, Qulsoom Hussain, Faisal Inayat, Muhammad Yasir, Rizwan Asim

**Affiliations:** 1 Department of Medicine, Shifa International Hospital, Islamabad, Pakistan; 2 Internal Medicine, Mercy Health Saint Mary's Grand Rapids; 3 Graduate, Shifa International Hospital, Islamabad, Pakistan; 4 Internal Medicine, Newark Beth Israel Medical Center; 5 Department of Medicine, Allama Iqbal Medical College, Lahore, Pakistan; 6 Internal Medicine, King Edward Medical University, Mayo Hospital, Lahore, Pakistan; 7 Internal Medicine, Texas Tech University (perian Basin)

**Keywords:** undifferentiated embryonal sarcoma of liver, uesl, chemotherapy, liver transplant, unresectable sarcoma

## Abstract

Undifferentiated embryonal sarcoma of the liver (UESL) is a malignancy of mesenchymal origin observed predominantly in the pediatric population and very rarely in adults. We describe the case of a 21-year-old male who presented with acute onset of right upper quadrant pain and distention. Physical examination of the patient revealed right upper quadrant tenderness with the lower border of the liver palpable, 4 cm below the right costal margin. Laboratory tests performed on admission showed that the patient’s liver function tests, urinalysis, complete blood count, and basic metabolic panel were within reference range. The levels of viral hepatitis and tumor serum markers were all within normal limits except for an elevated level of cancer antigen (CA) 19-9. Magnetic resonance imaging (MRI) and a computerized tomography (CT) scan showed two well-circumscribed lesions in the right lobe. The biopsy of the lesion showed UESL. The patient was started on chemotherapy. On his fifth cycle of chemotherapy, the patient was offered orthotopic liver transplantation (OLT). The patient underwent a successful OLT. There were no postoperative complications. Increased survival time and prevention of the recurrence of USEL can be achieved by surgical resection of the tumor combined with adjuvant and neoadjuvant chemotherapy. For unresectable tumors, OLT with chemotherapy can be a potential cure in younger patients.

## Introduction

Undifferentiated embryonal sarcoma of the liver (UESL) is a relatively rare tumor predominantly found in the pediatric population [1]. It is a very rare entity in adults, more commonly affecting females, and to the best of our knowledge, during the past 55 years, 50-60 cases in adults have been reported [2-4]. The presenting signs and symptoms are non-specific with patients usually presenting with abdominal pain, weight loss, liver mass, or fever [5]. UESL has a very poor prognosis with less than one year median survival time [1]. Surgical resection with adjuvant chemotherapy is considered to be the treatment of choice for resectable tumors to improve prognosis [6]. However, the current literature is considerably deficient regarding treatment of unresectable UESL. We describe a case of a 21-year-old male with unresectable UESL, his clinical course, and the use of chemotherapy as well as the importance of orthotopic liver transplantation (OLT) in unresectable UESL.

## Case presentation

 A 21-year-old male presented to our hospital with acute onset of right upper quadrant abdominal pain and distention. On abdominal examination, the patient had right upper quadrant tenderness and palpable liver 4 cm below the costal margin. No ascites, rebound tenderness, or guarding were found. No jaundice or jugular venous distention (JVD) were observed. The patient denied any history of fever, chills, or weight loss; however, he reported poor appetite. Initial investigations including blood counts, liver function tests, and a basic metabolic panel were within reference range. The patient had elevated cancer antigen (CA) 19-9 levels. His liver function test and serum tumor marker levels are shown in Table 1 and Table 2, respectively.

**Table 1:** Liver function tests (LFTs) ALT: alanine aminotransferase, AST: aspartate aminotransferase, ALP: alkaline phosphatase, INR: international normalized ratio

Tests	Results
Albumin (gram/deciliter)	4.3
ALT (IU/L)	31
AST (IU/L)	37
ALP (IU/L)	157
Bilirubin (milligram/deciliter)	1.0
INR	1.2

**Table 2:** Serum tumor markers CA: cancer antigen, CEA: carcinoembryonic antigen, HCG: human chorionic gonadotropin

Tests	Results
Lactic acid (micromole/liter)	0.6
Alpha Fetoprotein	1.2
CA 19-9	181 (normal<37)
CEA	0.8
HCG	1.9

**Figure 1:**
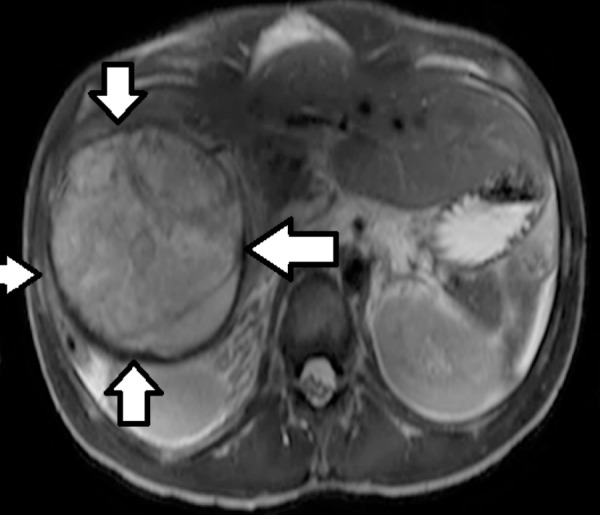
MRI showing cranial lesion of undifferentiated embryonal sarcoma of the liver MRI: Magnetic resonance imaging The cranial lesion of undifferentiated embryonal sarcoma of the liver has been highlighted by the arrows.

**Figure 2:**
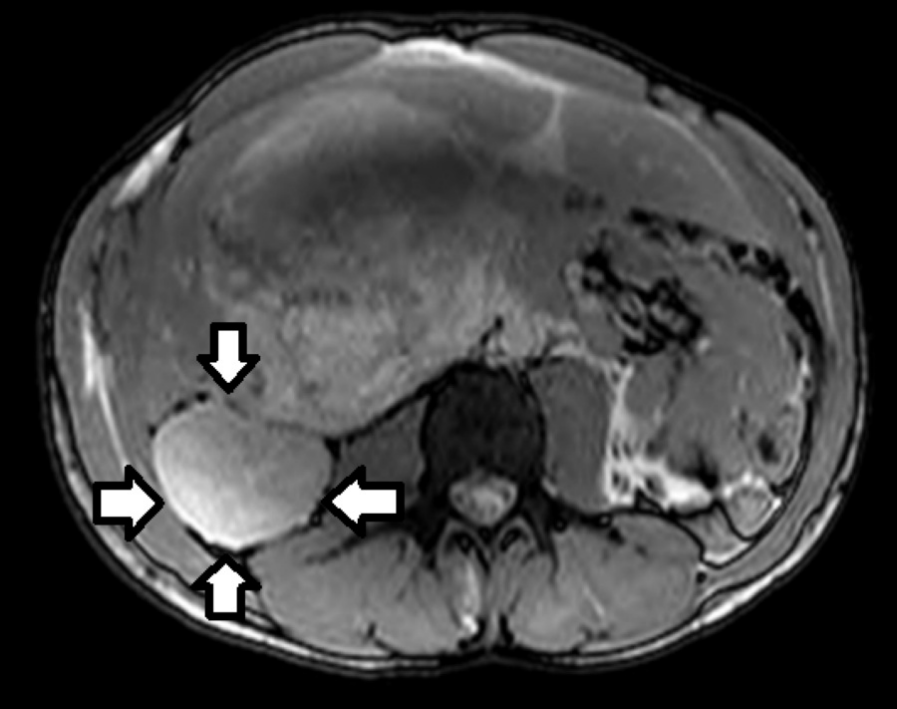
MRI showing caudal lesion of undifferentiated embryonal sarcoma of the liver MRI: Magnetic resonance imaging The caudal lesion of undifferentiated embryonal sarcoma of the liver has been highlighted by the arrows.

Abdominal imaging including magnetic resonance imaging (MRI) and a computerized tomography (CT) scan were performed. MRI showed two relatively well-circumscribed lesions, primarily located in the right lobe. The more cranial lesion measured 8.3 x 9.8 x 12.3 cm, while the more caudal mass measured 13.6 x 9.8 x 9.7 cm. There was no cirrhosis or biliary ductal dilatation. A small amount of perihepatic edema and fluid were observed. This is shown in Figure 1 and Figure 2.

The CT scan showed a space-occupying lesion in the right hepatic lobe, small right pleural effusion, and trace left pleural effusion. A fine needle biopsy was performed and the pathology report showed UESL. Based on imaging studies and biopsy reports, the tumor was found to be unresectable. The patient was started on ifosfamide and doxorubicin chemotherapy. During his fifth cycle of therapy, a donor's liver became available and he received an OLT. The explanted liver pathology showed 100% necrosis. The patient was followed by the outpatient department with regular CT-scan-monitoring over the course of 18 months. No lesions were seen on the CT scan. The patient had no postoperative complications.

## Discussion

Primary sarcoma of the liver is frequently found in children between the ages of six and ten [1]. It is a very rare clinicopathologic entity in adults. In adults, comparatively more cases in females have been reported as compared to males [4]. The median age of primary sarcoma of the liver was reported to be 25 years old in one study [6]. UESL accounts for less than 1% of primary sarcomas of the liver in adults, therefore metastatic sarcomas must first be excluded [4, 7]. The non-specific presentation of this tumor and the inconclusive radiological images make it a difficult clinical entity to diagnose. UESL usually presents as a solid, hyperechoic mass on ultrasound whereas CT scan and MRI images often show cystic lesions that are commonly mistaken for benign cystic tumors, which delays their diagnosis and, hence, affects their prognosis. Moreover, the histopathology of UESL is very poorly understood to date [8].

In adults, the prognosis for UESL was poor until recently when improvement in the survival and disease-free time period was achieved by using surgical resection, adjuvant chemotherapy, and/or OLT [9]. According to a study on 67 patients over the age of 15, it was concluded that patients who underwent complete surgical resection followed by adjuvant chemotherapy had a significantly better prognosis and median survival as compared to the patient who just underwent surgical resection [6]. According to some studies, the addition of neoadjuvant chemotherapy to surgical resection and adjuvant chemotherapy resulted in better prognoses [10]. The use of liver transplantation as a treatment modality for primary hepatic sarcoma in the adult is not clear. Some studies suggested OLT as an alternative to unresectable or refractory cases of UESL in the patient population younger than 21 years old [9].

In our case, the tumor was unresectable at the time of diagnosis due to its extensive local penetration. Chemotherapy with ifosfamide and doxorubicin was started to decrease the tumor burden. Liver transplantation potentially cured the disease in this case with no signs of recurrence after 18 months of follow-up.

## Conclusions

Increased survival time and prevention of the recurrence of UESL can be achieved by surgical resection of the tumor combined with adjuvant and neoadjuvant chemotherapy. For tumors that are not resectable, OLT with chemotherapy can be a potential cure in younger patients. The role of OLT in the treatment of primary liver sarcoma in adults is not clear and requires more research.
